# Effect of Metallothionein-III on Mercury-Induced Chemokine Gene Expression

**DOI:** 10.3390/toxics6030048

**Published:** 2018-08-12

**Authors:** Jin-Yong Lee, Maki Tokumoto, Gi-Wook Hwang, Min-Seok Kim, Tsutomu Takahashi, Akira Naganuma, Minoru Yoshida, Masahiko Satoh

**Affiliations:** 1 Laboratory of Pharmaceutical Health Sciences, School of Pharmacy, Aichi Gakuin University, 1-100 Kusumoto-cho, Chikusa-ku, Nagoya 464-8650, Japan; leejy@dpc.agu.ac.jp (J.-Y.L.); maki@dpc.agu.ac.jp (M.T.); 2 Laboratory of Molecular and Biochemical Toxicology, Graduate School of Pharmaceutical Sciences, Tohoku University, Sendai 980-8578, Japan; gwhwang@m.tohoku.ac.jp (G.-W.H.); assams7@naver.com (M.-S.K.); tsutomu@toyaku.ac.jp (T.T.); naganuma@tohoku.ac.jp (A.N.); 3 Laboratory Animal Center, Daegu-Gyeongbuk Medical Innovation Foundation, Daegu 41061, Korea; 4 Department of Environmental Health, School of Pharmacy, Tokyo University of Pharmacy and Life Sciences, 1432-1, Horinouchi, Hachioji, Tokyo 192-0392, Japan; 5 Faculty of Health and Medical Care, Hachinohe Gakuin University, 3-98 Mihono, Hachinohe 031-8588, Japan; m2yosida@hachinohe-u.ac.jp

**Keywords:** methylmercury, mercury vapor, metallothionein-III, chemokine

## Abstract

Mercury compounds are known to cause central nervous system disorders; however the detailed molecular mechanisms of their actions remain unclear. Methylmercury increases the expression of several chemokine genes, specifically in the brain, while metallothionein-III (MT-III) has a protective role against various brain diseases. In this study, we investigated the involvement of MT-III in chemokine gene expression changes in response to methylmercury and mercury vapor in the cerebrum and cerebellum of wild-type mice and MT-III null mice. No difference in mercury concentration was observed between the wild-type mice and MT-III null mice in any brain tissue examined. The expression of *Ccl3* in the cerebrum and of *Cxcl10* in the cerebellum was increased by methylmercury in the MT-III null but not the wild-type mice. The expression of *Ccl7* in the cerebellum was increased by mercury vapor in the MT-III null mice but not the wild-type mice. However, the expression of *Ccl12* and *Cxcl12* was increased in the cerebrum by methylmercury only in the wild-type mice and the expression of *Ccl3* in the cerebellum was increased by mercury vapor only in the wild-type mice. These results indicate that MT-III does not affect mercury accumulation in the brain, but that it affects the expression of some chemokine genes in response to mercury compounds.

## 1. Introduction

Several mercury compounds are considered hazardous, exerting mainly central nervous system (CNS) or renal damage [1,2,3]. Methylmercury is a pollutant that causes severe damage [4,5], and accumulates in fish by bioaccumulation. Recent epidemiological investigations have shown that pregnant women who take up relatively large amounts of methylmercury are at higher risk of delivering children with developmental disorders [6,7]. Mercury vapor also produces neuronal damage, and the exposure of gold miners to high levels of mercury vapor through mercury amalgamation is a great concern in developing countries [8,9]. Furthermore, the exposure of children to mercury vapor has increased due to the use of mercury in amalgam to extract gold from ores [10]. Recently, it was reported that high concentrations of methylmercury were detected in grains yielded near a mercury mine in China [11]. These reports suggest that rice is a source of methylmercury exposure in the area. In addition, the median estimated methylmercury intake for children was 0.29 µg/kg bodyweight/week, which is approximately 16% above the dietary references dose (RfD) in Hong Kong [12]. Furthermore, a recent Japanese cohort study suggested that prenatal methylmercury exposure affected neuronal function during child development [13,14].

Metallothionein (MT) is a defense factor against harmful metals such as mercury and cadmium. MT is a cysteine rich, low molecular protein that reduces toxicity by binding to metals [15,16]. There are four isoforms of MT, I to IV, which have different locations and levels of expression and varied physiological functions [15,16]. MT-III, which is present in the brain, has a protective effect against various brain diseases [17,18,19].

Recently, it has been reported that methylmercury increases the expression of several chemokine genes specific to the mouse brain [20]. Furthermore, we examined the impact of methylmercury on the expression of chemokine genes in various mouse tissues and found that expression of *Ccl4* shows brain specific induction by methylmercury treatment [21]. Chemokines are a type of cytokine known to cause migration of leukocytes. They are secreted primarily from immune cells and participate in the inflammatory response [22]. Chemokines are also secreted from various tissues, including the brain, kidney, and liver [23,24,25,26].

In this study, we investigated the involvement of MT-III in chemokine gene expression in the cerebrum and cerebellum in response to methylmercury and mercury vapor using the MT-III null mice. 

## 2. Materials and Methods

### 2.1. Animals and Exposure Procedures

MT-III null mice and 129/Sv mice as wild-type controls were purchased from The Jackson Laboratory (Bar Harbor, ME, USA) and routinely bred in the vivarium of the School of Pharmacy, Aichi Gakuin University. MT-III null mice were engineered by Erickson et al. [18] and had the 129/Sv genetic background. Three-week-old female mice were caged in a ventilated animal room at 24 °C ± 1 °C with 50 ± 10% relative humidity, and a 12 h light–dark cycle in the animal room of the School of Pharmacy, Aichi Gakuin University. Mice were maintained on standard laboratory food (MF, Oriental Yeast Co., Tokyo, Japan) and tap water ad libitum, and they received humane care throughout the experiment according to the guidelines of the School of Pharmacy.

Mice were assigned randomly to control or experimental groups (n = 4–5). For mercury vapor (Hg^0^) exposure, mice were placed in a mercury vapor exposure chamber and exposed for 8 h every day at a mean concentration of 0.121 (range: 0.080 to 0.180) mg/m^3^ for 4 weeks. The concentration of mercury in the exposure chamber was measured every day using a mercury survey meter (EMP-1A, Nippon Instruments Co., Tokyo, Japan). For methylmercury (CH_3_Hg^+^) exposure, methylmercury chloride (GL Sciences Inc., Tokyo, Japan) was diluted with distilled water to prepare a 5 ppm solution. The solution containing 5 ppm methylmercury was given ad libitum instead of tap water. After 4 weeks of exposure, the cerebrum and cerebellum were removed from each mouse under ether anesthesia.

### 2.2. Real-Time Reverse Transcription-Polymerase Chain Reaction (RT-PCR)

Total RNA was extracted from brain tissues using TRIzol^®^ Reagent (Ambion, Grand Island, NY, USA) according to the manufacturer’s instructions. Total RNA was incubated with a PrimeScript™ RT Reagent Kit (Perfect Real Time) (TaKaRa Bio, Shiga, Japan) to generate cDNA. Real time PCR was performed using SYBR Premix Ex Taq™ II (Perfect Real Time) (TaKaRa Bio) and a Thermal Cycler Dice Real time system (TaKaRa Bio). PCR conditions were: 10 s of hot start at 95 °C followed by 40 cycles of 5 s at 95 °C and 30 s at 60 °C. Gene expression was normalized to *β-actin* mRNA levels. Oligonucleotide sequences of the primers (sense and antisense, respectively) were: 5′-TCTAAGCGTCACCACGACTTCA-3′ and 5′-GTGCACTTGCAGTTCTTGCAG-3′ for the mouse *MT-I* gene; 5′-CCTGCAATGCAAACAACAATGC-3′ and 5′-AGCTGCACTTGTCGGAAGC-3′ for the mouse *MT-II* gene; 5′-AGGGCTGCAAATGCACG-3′ and 5′-ACACACAGTCCTTGGCACACTTC-3′ for the mouse *MT-III* gene; 5′-ATGAAGGTCTCCACCACTGC-3′ and 5′-CCCAGGTCTCTTTGGAGTCA-3′ for the mouse *Ccl3* gene; 5′-CAAACCTAACCCCGAGCAACAC-3′ and 5′-GGTCTCATAGTAATCCATCACAAAGC-3′ for the mouse *Ccl4* gene; 5′-AATGCATCCACATGCTGCTA-3′ and 5′-CTTTGGAGTTGGGGTTTTCA-3′ for the mouse *Ccl7* gene; 5′-GTCCTCAAGGTATTGGCTGGA-3′ and 5′-GGGTCAGCACAGATCTCCTT-3′ for the mouse *Ccl12* gene; 5′-AAGTGCTGCCGTCATTTTCT-3′ and 5′-GTGGCAATGATCTCAACACG-3′ for the mouse *Cxcl10* gene; 5′-CCTAAGGCCAACCGTGAAAA-3′ and 5′-AGGCATACAGGGACAGCACA-3′ for the mouse *β-actin* gene.

### 2.3. Analysis of Mercury Concentrations in Tissues

Mercury concentrations in tissues were measured with a cold vapor atomic absorption spectrophotometer (RA-3 Mercury Analyzer; Nippon Instruments, Tokyo, Japan) after digestion with a concentrated acid mixture [HNO_3_/HClO_4_ 1:3 (*v*/*v*)].

### 2.4. Statistical Analyses

Statistical analyses were undertaken using single factor ANOVA followed by Bonferroni’s test for post hoc comparison (*P* < 0.05).

## 3. Results

### 3.1. Body Weight Changes

**Figure 1:**
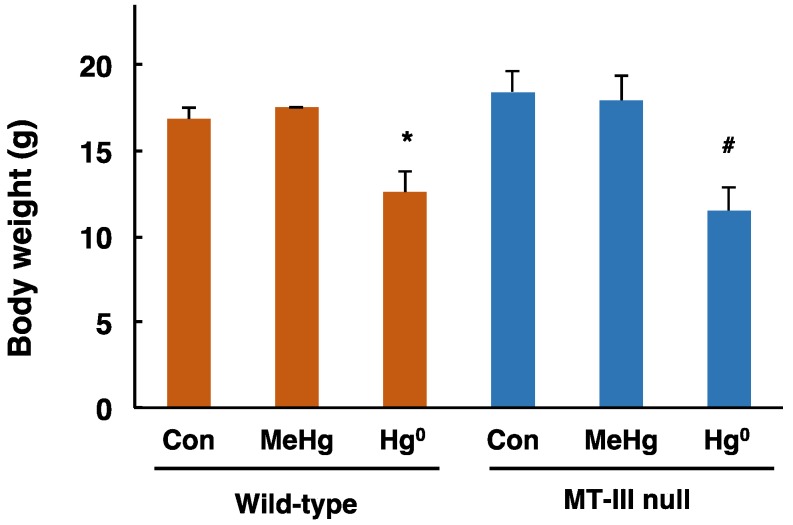
Body weights of the wild-type and MT-III null mice exposed to mercury compounds. Body weights of the wild-type and MT-III null mice were measured one day after the final mercury exposure. Values are the mean ± S.D. (n = 4–5). * Significantly different from the control group of wild-type mice, *P* < 0.05. ^#^ Significantly different from the control group of MT-III null mice, *P* < 0.05.

Body weights of the MT-III null mice and wild-type mice were measured one day after completion of exposure. The body weights of wild-type mice and MT-III null mice exposed to methylmercury were similar to those of the corresponding control mice (Figure 1). The body weights of wild-type mice and MT-III null mice exposed to mercury vapor were significantly lower than those of the corresponding control mice (Figure 1). However, there was no difference in body weight fluctuation in response to mercury exposure between wild-type mice and MT-III null mice (Figure 1). 

### 3.2. Total Mercury Concentrations in the Cerebrum and Cerebellum

**Figure 2:**
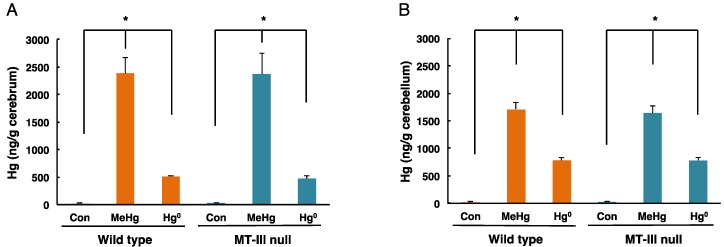
Mercury concentrations in the cerebrum and cerebellum of wild-type mice and MT-III null mice exposed to mercury compounds. Cerebra and cerebella were removed one day after the final mercury exposure. Total mercury levels in the cerebrum (**A**) and cerebellum (**B**) were measured. Values are the mean ± S.D. (n = 4–5). * *P* < 0.05.

The total mercury levels in the cerebrum and cerebellum of mice exposed to methylmercury or mercury vapor are shown in Figure 2. In the cerebrum of wild-type mice, mercury concentrations of 2380.57 ± 300.24 ng/g tissue and 519.99 ± 13.68 ng/g tissue were detected after methylmercury exposure and mercury vapor exposure, respectively (Figure 2A). In the cerebrum of MT-III null mice, mercury concentrations of 2373.14 ± 957.12 ng/g tissue and 475.79 ± 56.73 ng/g tissue were detected after methylmercury exposure and mercury vapor exposure, respectively (Figure 2A). In the cerebellum of wild-type mice, mercury concentrations of 1715.23 ± 121.09 ng/g tissue and 783.58 ± 44.15 ng/g tissue were detected following methylmercury exposure and mercury vapor exposure, respectively (Figure 2B). In the cerebellum of MT-III null mice, mercury concentrations of 1643.18 ± 130.82 ng/g tissue and 779.71 ± 54.85 ng/g tissue were detected in response to methylmercury exposure and mercury vapor exposure, respectively (Figure 2B). However, no significant difference was observed between wild-type mice and MT-III null mice in either the cerebrum or cerebellum (Figure 2). 

### 3.3. The Levels of MT-I, MT-II, MT-III mRNAs in the Cerebrum and Cerebellum after Mercury Exposure

**Figure 3:**
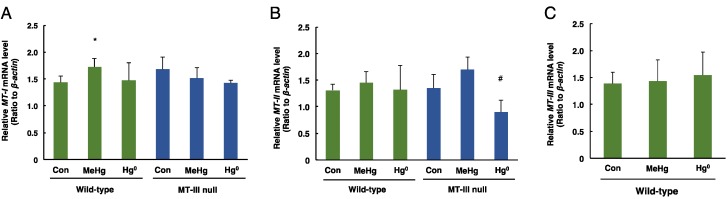
The levels of *MT-I*, *MT-II* and *MT-III* mRNAs in the cerebra of wild-type mice and MT-III null mice exposed to mercury compounds. mRNA levels of *MT-I* (**A**), *MT-II* (**B**) and *MT-III* (**C**) were determined by real time RT-PCR. mRNA levels were normalized with *β-actin*. Values are the mean ± S.D. (n = 4–5). * Significantly different from the control group of wild-type mice, *P* < 0.05. ^#^ Significantly different from the control group of MT-III null mice, *P* < 0.05.

The mRNA levels of *MT-I* in the cerebrum were significantly increased only in the wild-type mice exposed to methylmercury (Figure 3A). The mRNA levels of *MT-II* in the cerebrum were significantly decreased only in MT-III null mice exposed to mercury vapor (Figure 3B). The *MT-III* mRNA levels in the cerebrum of wild-type mice were not altered by methylmercury or mercury vapor exposure (Figure 3C).

**Figure 4:**
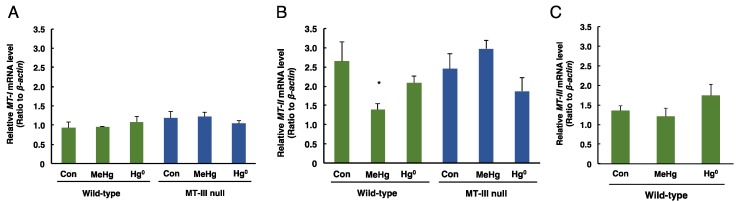
The levels of *MT-I*, *MT-II* and *MT-III* mRNAs in the cerebella of wild-type mice and MT-III null mice exposed to mercury compounds. mRNA levels of *MT-I* (**A**), *MT-II* (**B**) and *MT-III* (**C**) were determined by real time RT-PCR. mRNA levels were normalized with *β-actin*. Values are the mean ± S.D. (n = 4–5). * Significantly different from the control group of wild-type mice, *P* < 0.05.

The mRNA levels of *MT-I*, *MT-II* and *MT-III* in the cerebellum of each exposed group are shown in Figure 4. *MT-I* mRNA levels in the cerebellum were not changed by mercury exposure in either wild-type mice or MT-III null mice (Figure 4A). The mRNA levels of *MT-II* in the cerebellum were significantly decreased only in the wild-type mice exposed to methylmercury (Figure 4B). No changes in *MT-III* mRNA levels were observed in the wild-type mice exposed to methylmercury or mercury vapor (Figure 4C). These results indicate that MT-III does not affect the accumulation of mercury after methylmercury or mercury vapor exposure.

### 3.4. Changes in Expression of Chemokine Genes in the Cerebrum in Response to Mercury Exposure

**Figure 5:**
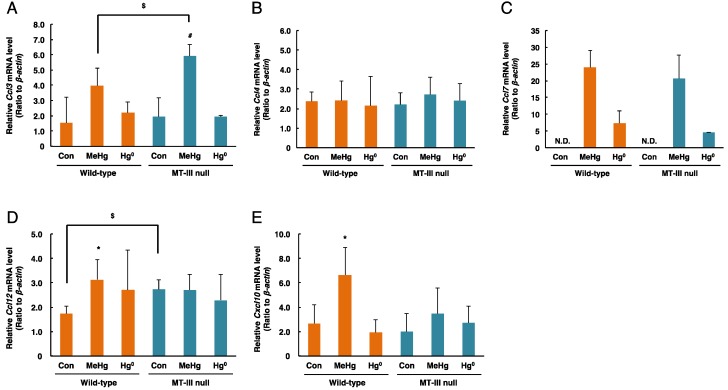
mRNA levels of chemokine genes in the cerebra of wild-type mice and MT-III null mice exposed to mercury compounds. mRNA levels of *Ccl3* (**A**), *Ccl4* (**B**), *Ccl7* (**C**), *Ccl12* (**D**) and *Cxcl10* (**E**) were determined by real-time RT-PCR. mRNA levels were normalized with *β-actin*. Values are the mean ± S.D. (n = 4–5). * Significantly different from the control group of wild-type mice, *P* < 0.05. ^#^ Significantly different from the control group of MT-III null mice, *P* < 0.05. ^$^ Significantly different between wild-type and MT-III null mice, *P* < 0.05.

*Ccl3* mRNA levels in the cerebrum were significantly increased by methylmercury exposure only in the MT-III null mice (Figure 5A). Moreover, *Ccl3* mRNA levels in the cerebrum of MT-III null mice were higher than those of wild-type mice by methylmercury exposure (Figure 5A). *Ccl4* mRNA levels did not change after mercury exposure (Figure 5B). *Ccl7* mRNA levels were markedly elevated in the MT-III null mice and wild-type mice after methylmercury and mercury vapor exposure (Figure 5C). Although *Ccl12* mRNA levels were increased by MT-III deficiency, methylmercury exposure increased *Ccl12* mRNA levels only in the wild-type mice (Figure 5D). *Cxcl10* mRNA levels were significantly elevated only when the wild-type mice were exposed to methylmercury (Figure 5E).

### 3.5. Changes in Expression of Chemokine Genes in the Cerebellum after Mercury Exposure

**Figure 6:**
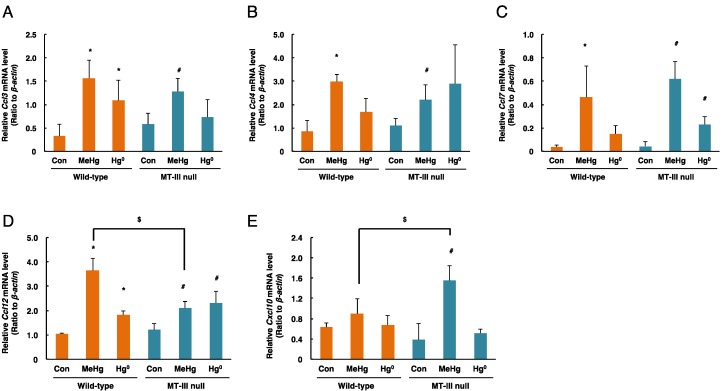
mRNA levels of chemokine genes in the cerebella of wild-type mice and MT-III null mice exposed to mercury compounds. mRNA levels of *Ccl3* (**A**), *Ccl4* (**B**), *Ccl7* (**C**), *Ccl12* (**D**) and *Cxcl10* (**E**) were determined by real-time RT-PCR. mRNA levels were normalized with *β-actin*. Values are the mean ± S.D. (n = 4–5). * Significantly different from the control group of wild-type mice, *P* < 0.05. ^#^ Significantly different from the control group of MT-III null mice, *P* < 0.05. ^$^ Significantly different between wild-type and MT-III null mice, *P* < 0.05.

The mRNA levels of *Ccl3* in the cerebellum were significantly increased by methylmercury in the MT-III null mice and wild-type mice, and mercury vapor significantly increased *Ccl3* mRNA levels only in the wild-type mice (Figure 6A). *Ccl4* mRNA levels were significantly increased by methylmercury in the MT-III null mice and wild-type mice (Figure 6B). *Ccl7* mRNA levels were significantly increased by methylmercury in the MT-III null mice and wild-type mice, and were significantly increased only in the MT-III null mice in response to mercury vapor (Figure 6C). *Ccl12* mRNA levels were significantly increased by both methylmercury and mercury vapor in the MT-III null mice and wild-type mice (Figure 6D). However, *Ccl12* mRNA levels in the MT-III null mice exposed to methylmercury were lower than those of wild-type mice (Figure 6D). *Cxcl10* mRNA levels were significantly elevated only when the MT-III null mice were exposed to methylmercury (Figure 6E).

## 4. Discussion

MT-III is highly expressed in neurons [17], and also shows a protective effect against brain diseases [18,19]. Mercury compounds, such as methylmercury and mercury vapor, are harmful substances that cause disorders of the CNS, but the influence of MT-III on the disorders caused by these mercuric compounds is poorly understood. Recently, methylmercury has been reported to increase expression of the chemokine genes, *Ccl3*, *Ccl4*, *Ccl7*, *Ccl12* and *Cxcl10* in the mouse brain [20,21,27]. The most highlighted knowledge in this study is that *Ccl3* expression in the cerebrum and the cerebellar expression of *Ccl12* and *Cxcl10* were significantly different between wild-type mice and MT-III null mice only in the methylmercury exposure. MT-III is abundantly expressed in the normal brain; on the other hand, in a brain with Alzheimer’s disease, MT-III expression is largely reduced [17]. It was suggested that MT-III might have a protective role in cerebral ischemia [28]. Furthermore, inflammatory factors are upregulated upon the brain damage, as it increases phagocytosis and the release of inflammatory mediators [29,30]. The present study suggests that the increase in chemokine expression may be involved in methylmercury-triggered brain damage with topical specificity. The present study also suggest that MT-III may be the cause of the difference in inflammatory response upon methylmercury exposure.

Our present study revealed that the MT-III deficiency had no effect on mercury concentration in the brains of mice exposed to mercury compounds; however, the MT-III deficiency influenced the expression of chemokine genes in response to mercury exposure. The expression of *Ccl12* and *Cxcl10* in the cerebrum was increased in the wild-type mice by methylmercury; but not in the cerebrum of MT-III null mice. Moreover, in response to mercury vapor exposure, *Ccl3* expression in the cerebellum was not changed in the MT-III null mice, but was significantly elevated in the wild-type mice. In the cerebellum of wild-type mice, the expression of *Ccl7* was not changed by mercury vapor exposure and that of *Cxcl10* was not changed by methylmercury exposure; however, they were significantly elevated in the MT-III null mice. Indeed, the fluctuation in *Cxcl10* expression in response to methylmercury was observed in the cerebrum of wild-type mice, and in the cerebellum of MT-III null mice. Taken together, these findings indicate that MT-III may affect the expression of some chemokine genes in response to different mercury compounds differently in the cerebrum and cerebellum. In recent years, MT-I and MT-II have been reported to be involved in inflammatory reactions in cardiomyocytes [31]. The present study indicates that MT-III may be involved in inflammatory reactions consequent to an exogenous stress on the brain. 

Chemokines are associated with inflammatory damage in a number of tissues, including the brain [23,24,25,26]. Chemokines may function as signaling molecules in the CNS [32]. Accordingly, the expression of several chemokines increases with hypoglossal nerve damage, however the role of chemokines in cranial nerves remains unclear. Methylmercury induces a brain-specific increase in *Ccl4* expression in mice [21]. In addition, it is assumed that CCL4 is involved in the pathway mediating the toxicity of methylmercury in the CNS [33,34]. In this study, methylmercury induced *Ccl4* gene expression in the cerebellum not only in the wild-type mice but also in the MT-III null mice. Therefore, CCL4 may be an important factor mediating methylmercury toxicity. 

Previous studies revealed that postnatal mice exposed to 0.188 mg/m^3^ mercury vapor or 3.85 ppm methylmercury showed a decrease in total locomotive activity in the OPF [35]. In this study, we exposed the mice to similar doses of mercury compounds. Because several chemokine gene expressions were increased in brain tissues of wild-type mice, chemokine genes may be associated with the influence of mercury exposure in childhood on neuronal development. 

MT-I/II null mice showed an enhanced adverse effect of neonatal mercury (HgCl_2_) exposure [19]. Moreover, MT-I/II null mice exposed to mercury vapor showed decreased locomotor activity [36,37]. Furthermore, the highly sensitive methylmercury toxicity in MT-I/II deficient astrocytes was rescued by the introduction of the MT-I gene [38]. Thus, MT-I and MT-II were suggested to be involved in mercury compound induced neuropathy, but the role of MT-III in neuropathy caused by mercury compounds is almost unknown. Our present study revealed that MT-III deficiency is involved in mercury-induced neuropathy through changing cerebral chemokine gene expression.
